# A Novel Approach to extrude Subgingivally Fractured Tooth using Customized Removable Appliance

**DOI:** 10.5005/jp-journals-10005-1484

**Published:** 2017-02-01

**Authors:** Ziauddin Mohammad, Sarada Penmetcha, Apeksha Bagalkotkar, Srinivas Namineni

**Affiliations:** 1 Associate Professor, Department of Pedodontics and Preventive Dentistry, Sri Sai College of Dental Surgery, Hyderabad, Telangana, India; 2 Professor, Department of Pedodontics and Preventive Dentistry, Sri Sai College of Dental Surgery, Hyderabad, Telangana, India; 3 Senior Lecturer, Department of Pedodontics and Preventive Dentistry, Sri Sai College of Dental Surgery, Hyderabad, Telangana, India; 4 Associate Professor, Department of Pedodontics and Preventive Dentistry, Sri Sai College of Dental Surgery, Hyderabad, Telangana, India

**Keywords:** Begg bracket, Complicated crown root fracture, Fiber post, Orthodontic extrusion, Rolled cone technique.

## Abstract

Traumatic dental injuries (TDIs) are common in growing children. Among all the dental injuries, complicated crown root fractures (Andreasen Class VI) of maxillary permanent anterior teeth are relatively widespread. Such fractured teeth are often considered as hopeless and are extracted. However, if the tooth is to be retained, various treatment strategies have been proposed. The aim of the present case report is to suggest a new technique to treat a complicated crown root fracture. The management of this case included endodontic procedure and orthodontic extrusion to move the fracture line above the supragingival level. A customized removable Hawley’s appliance with a modified single cantilever spring was fabricated and an anchoring Begg bracket was bonded on the residual crown of the tooth. This method is useful in the mixed dentition when there is insufficient anchorage of adjacent teeth because of preshedding mobility and trauma.

**How to cite this article:** Mohammad Z, Penmetcha S, Bagalkotkar A, Namineni S. A Novel Approach to extrude Subgingivally Fractured Tooth using Customized Removable Appliance. Int J Clin Pediatr Dent 2018;11(1):53-57.

## INTRODUCTION

Trauma with an accompanying fracture of a permanent incisor is a tragic experience for the young patient and it is also a problem, the management of which requires knowledge, judgment, and expertise. Therefore, it is possibly unique by any other portion of the dentist’s practice. Trauma to the oral region occurs frequently and comprises 5% of all injuries for which people seek treatment. Among all the facial injuries, dental injuries are the most frequently reported, usually occurring around 8 to 12 years of age.^1^ Out of the reported dental injuries, around 70% involve the maxillary central incisors followed by maxillary lateral incisors and mandibular incisors.^2^

Complicated crown root fractures involve the enamel, dentin, and part of the root (cementum) surface of the tooth. The fracture line often passes subgingivally or crests of the alveolar bone, presenting a very difficult situation for restorations. Such fractured teeth are often considered as hopeless and are extracted.^3^ However, if the tooth is to be retained, the orthodontic slow extrusion is one of the alternative treatment options. The goal of extrusion is to obtain a crown root ratio of approximately 1:1. A central incisor can be extruded 2 to 4 mm, while a lateral incisor can be 4 to 6 mm.^4^ In the present case, a customized removable Hawley’s appliance with a modified single cantilever spring was fabricated and an anchoring Begg bracket was bonded on the remaining crown of the tooth. This method is useful when the adjacent teeth are mobile or offer inadequate anchorage because of preshedding mobility, transient dentition, and trauma when mild force is required.

## CASE REPORT

**Figs 1A and B:**
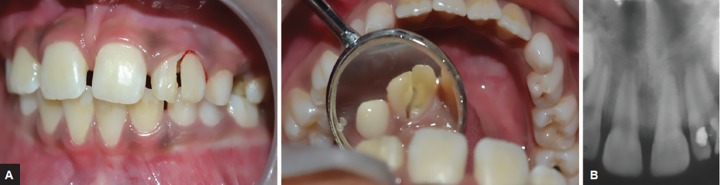
(A) Intraoral photographs: the fracture line extending from the incisor edge to below the free gingival margin involving both labial and palatal surfaces of maxillary left lateral incisor. (B) Intraoral periapical radiograph: the fracture line extending at the level of crest of alveolar bone

A 10-year-old boy reported to the Department of Pedodontics and Preventive Dentistry, Sri Sai College of Dental Surgery, Hyderabad, Telangana, India, with a chief complaint of pain and mobility in the upper front tooth and a history of bicycle accident 1 week earlier. His medical history and extraoral examination were satisfactory. Intraoral examination revealed an oblique fracture in the maxillary left lateral incisor, the fracture line extending from the incisor edge to below the free gingival margin involving both labial and palatal surfaces, and the pulp exposure was observed (Fig. 1A). The fracture segment was mobile and attached to the gingival fibers and the tooth was tender.

Radiographic examination confirmed the clinical findings. Intraoral periapical radiograph showed the fracture line on labial and the palatal side and could be traced at the level of the alveolar crest. The space between the residual crown and the fractured segment was evident, and an incomplete root apex of left maxillary lateral incisor was seen (Fig. 1B). The periodontal ligament space around the tooth was widened; there were no associated injuries to the adjacent teeth. Based on clinical and radiographic findings, the diagnosis was confirmed as complicated crown root fracture of maxillary left lateral incisor (Andreasen Class VI). Informed consent was taken from the parents after explaining the benefits, risks, duration, and costs of preserving the maxillary left lateral incisor using orthodontic extrusion.

**Figs 2A and B:**
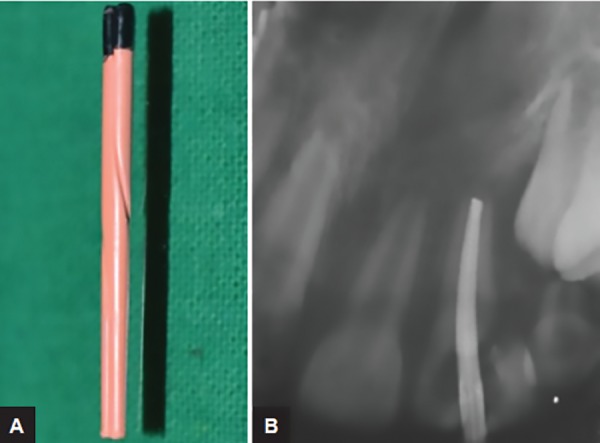
Obturation with gutta-percha roll cone technique

## ENDODONTIC PROCEDURE

Local anesthesia was administrated prior to the procedure. (LIGNOX 2% A, adrenaline, Lignocaine 1: 80000, Lic No: 557, Indoco remedies Ltd). The access cavity preparation and pulp extirpation were done and the root canal was filled with intracanal triple antibiotic paste 3 Mix (ciprofloxacin, metronidazole, and minocycline). The access cavity was sealed with zinc oxide eugenol cement. The second visit of the patient was scheduled after a week. The canal was obturated with gutta-percha (Pearl Endopia, Pear Dent Co., Ltd. Korea) rolled cone technique (Fig. 2) and Endomethasone sealer was used (Septodont, Saint-Maur-des-Fosses, France). Finally, the access cavity was sealed with glass ionomer cement (GIC), and the fractured fragment was removed. Four weeks after the root canal obturation, the patient was scheduled for further treatment. As the fracture line extended sub-gingivally, at the level of the alveolar crest, orthodontic extrusion was considered as the treatment of choice to establish a good cervical finish line above the supra gingival margin before the final restoration.

## APPLIANCE DESIGN

**Figs 3A and B:**
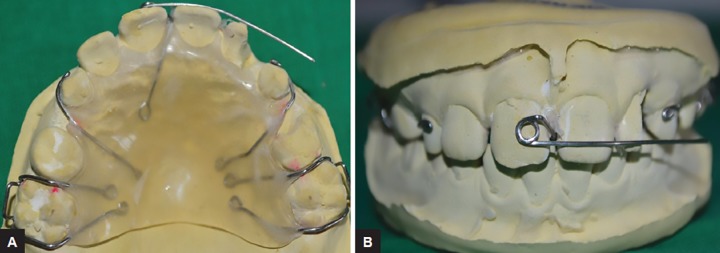
Customized removable Hawley’s appliance with modified single cantilever spring design

An alginate impression was made and the working cast was prepared. The Adams clasps were made on maxillary permanent first molars and C clasp on primary canines with a 21-gauge round stainless steel wire (Konark, Ever bright dental, Khokhar, India). The modified single cantilever spring (23-gauge round stainless steel wire) was made, the retention arm toward the palatal side, the active arm perpendicular to the long axis of incisors, placing on the labial side at the junction of middle and incisal third of crown of centrals, and a 3 mm internal diameter of helix was placed on the labial surface of the maxillary permanent central incisor. The clasp and single cantilever spring were stabilized with self-cure acrylic resin (Fig. 3).

## ORTHODONTIC EXTRUSION

**Figs 4A and B:**
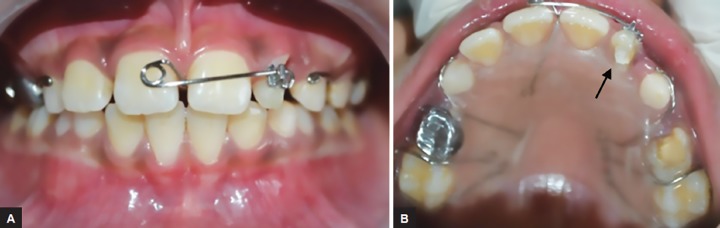
Customized removable Hawley’s appliance with modified single cantilever spring, arrow showing cervical contour of acrylic plate to prevent unwanted axial movement of 22

A customized removable Hawley’s appliance with modified single cantilever spring was inserted in the patient’s mouth and an anchoring Begg bracket (Classic Orthodontics, USA) was bonded on the residual crown of the maxillary left lateral incisor. The single cantilever spring active arm was engaged in the vertical bracket slot to ensure that the line of action of the force stays along the long axis of the tooth and palatal acrylic plate was trimmed according to the cervical contour of 22 to prevent unwanted axial movements (Fig. 4). In order to prevent the occlusal interference during activation, the bite is opened with a GIC block of 1 mm on the occlusal surface of mandibular molars. The patient was instructed to wear the appliance properly and engage the active arm in the vertical bracket slot.

**Fig. 5:**
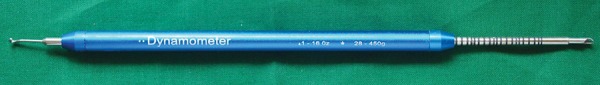
Dontrix gauge (Dynamometer)

**Figs 6A and B:**
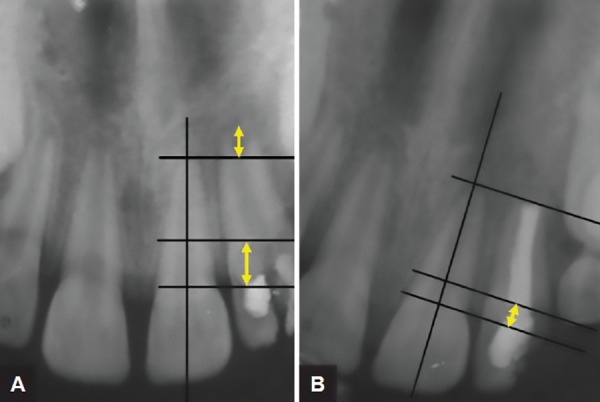
(A) Before and (B) after extrusion of maxillary left lateral incisor

**Figs 7A and B:**
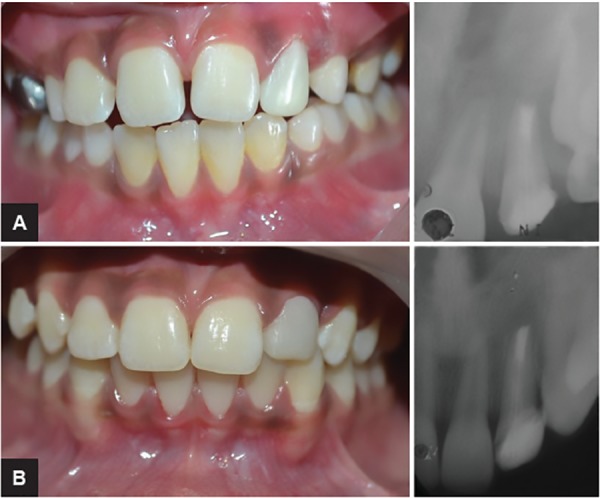
(A) With polycarbonate provisional crown 22, (B) composite restoration

**Figs 8A to D:**
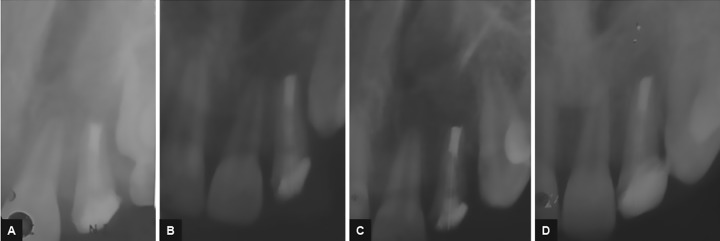
Intraoral periapical radiographs: (A) First year; (B) second year; (C) third year; and (D) fourth year follow-up

A periodic activation (opening of the helix) was done at weekly intervals; the force was measured with a Dontrix gauge (Fig. 5) to deliberate the optimal force (20 gm).^5^ The tooth was extruded about 4 mm at the end of 15 weeks, following retention with the same appliance for another 4 weeks without activation to retain the tooth in the extruded position. The extrusion was acceptable for the optimal cervical seal, before and after extrusion of maxillary left lateral incisor as shown (Fig. 6). The Hawley’s appliance and Begg bracket were removed and coronal gutta-percha was removed with a Peeso reamer (32 mm, #2, LOT P100912100, MANI, JAPAN) and a 5 mm of gutta-percha was left apically. Then, the root canal was dried with absorbent paper points (Sure endo, Sure Dent Corporation, LOT P444M, Jungwon gu, Korea) and the canal space was acid etched (Ezee etch-37, etchant gel, mission dental, EA08, USA). The fiber optic post (Reforpost, Angelus, E633127,

Brazil) was cemented with self-adhesive universal resin cement (RelyX U200, 3M ESPE, Germany) and core build-up was done with a composite restoration (3M ESPE, Filtek Z350 XT, USA). The tooth was restored with polycarbonate crown initially; later on, composite restoration was given (Fig. 7) and periodically followed up to 4 years (Fig. 8).

## DISCUSSION

A number of treatment alternatives are available depending on the position and circumferential extent of the fracture and the severity of the fracture in a subgingival direction. The treatment options were reviewed by Mule and Heithersay and they include periodontal surgery to expose the crown margins for better cervical restoration.^6-8^ Although the treatment of the crown-root fracture can be complex and time consuming, most teeth with these types of fractures can be saved. Extrusion is the easiest orthodontic movement to achieve, as it closely mimics natural tooth eruption.^9^

The extrusion involves applying traction forces in all areas of the periodontal ligament to stimulate marginal apposition of crestal alveolar bone. As the periodontal fibers are attached to the root cementum, during the extrusion process, the gingiva follows the vertical movement of the root. In the same way, the alveolar process which is attached to the root by periodontal ligament fibers is pulled along by the movement of the root.^2^ The slow extrusion meets all the criteria, and the alveolar bone surrounding the root will move along the tooth. It is essential that the constant slow force be maintained between the extrusion and hyalinization phases; otherwise, the desired orthodontic movement will not occur.^10^ By using slow orthodontic extrusion, the surgical exposure could be avoided, as it involves additional resection of bone surrounding the tooth.^7^ The adverse effect of rapid extrusion is associated with the periodontal ligament rupture and the tooth may get ankylosed, and in the extreme situation, it can also lead to root resorption.^11,12^

A force of 20 to 30 gm is an optimal orthodontic force for single rooted tooth; the applied force should be based on the physiologic response of individual tooth depending on its root size, root length, root morphology, and periodontal support.^7^ Based on the rate of tooth movement, 1 mm of extrusion per week is considered physiologic for slow extrusion.^5^ In the present case, a 20 gm of force was applied and the tooth moved at a very slow rate of 1 mm/5 weeks; it was determined to keep the force level minimum to bring about extrusion without damage to the supporting tissues and root. Even though the recall visits are more and time consuming with removable appliance, the technique used in this case is very simple with minimum inventory, cost effective, and the results are acceptable. More clinical research is required with a larger sample to assess the success of the appliance.

## CONCLUSION

The orthodontic slow extrusion is one of the treatment options for subgingivally fractured teeth, as it meets all the physiological tooth movement criteria. The restoration of the fractured anterior teeth not only improves the appearance of the child, but also significantly reduces the psychological impact.
